# Multi-Cat Monitoring System Based on Concept Drift Adaptive Machine Learning Architecture

**DOI:** 10.3390/s23218852

**Published:** 2023-10-31

**Authors:** Yonggi Cho, Eungyeol Song, Yeongju Ji, Saetbyeol Yang, Taehyun Kim, Susang Park, Doosan Baek, Sunjin Yu

**Affiliations:** 1 Research and Development Department, Codevision Inc., Seoul 03722, Republic of Korea; cynki@codevision.kr (Y.C.); song@codevision.kr (E.S.); jyj9802@codevision.kr (Y.J.); mindstar@codevision.kr (S.Y.); 2 Development Department, Valiantx Co., Ltd., Bucheon 14553, Republic of Korea; kim@purrit.net (T.K.); sspark@purrit.net (S.P.); ds@purrit.net (D.B.); 3 Department of Culture Techno, Changwon National University, Changwon 51140, Republic of Korea

**Keywords:** computer vision, machine learning, cat identification, animal monitoring, model retraining

## Abstract

In multi-cat households, monitoring individual cats’ various behaviors is essential for diagnosing their health and ensuring their well-being. This study focuses on the defecation and urination activities of cats, and introduces an adaptive cat identification architecture based on deep learning (DL) and machine learning (ML) methods. The architecture comprises an object detector and a classification module, with the primary focus on the design of the classification component. The DL object detection algorithm, YOLOv4, is used for the cat object detector, with the convolutional neural network, EfficientNetV2, serving as the backbone for our feature extractor in identity classification with several ML classifiers. Additionally, to address changes in cat composition and individual cat appearances in multi-cat households, we propose an adaptive concept drift approach involving retraining the classification module. To support our research, we compile a comprehensive cat body dataset comprising 8934 images of 36 cats. After a rigorous evaluation of different combinations of DL models and classifiers, we find that the support vector machine (SVM) classifier yields the best performance, achieving an impressive identification accuracy of 94.53%. This outstanding result underscores the effectiveness of the system in accurately identifying cats.

## 1. Introduction

The increasing trend of households choosing to adopt pet cats (*Felis catus*) is evident in modern society. The surge in cat ownership has brought about greater awareness and concern for the well-being and overall healthcare of these feline companions. This growing interest has, in turn, led to a spike in scientific and veterinary research aimed at proper diagnosis, health management, and treatment of diseases pertaining to cats [1,2,3,4]. Nevertheless, as the emphasis on cat healthcare increases, specific challenges have arisen, particularly in settings where multiple cats coexist. For households with more than one cat or in locations such as cat shelters, closely monitoring the health and well-being of each individual cat has become a considerably more complex undertaking. Each cat possesses unique personalities, behaviors, and health profiles, necessitating additional attention and diligence in their monitoring. In addition to ensuring that the cats are fed and sheltered, it is also crucial to spot any subtle signs of health issues, which can often go unnoticed in a multi-cat environment.

Consequently, systems for remote monitoring in multi-cat households are continuously being researched. For instance, Majid et al. [5] studied an IoT-based cat feeding and monitoring system, identifying which cat ate the food through RFID tags attached to the cats’ collars. Eagan et al. [6] researched a computer vision-based marker tracking system for cats in shelters, tracking the behavior of individual cats. They identified the cats by detecting 2D ArUco markers printed on paper collars attached to the cats’ necks. While these contact-based systems can continuously monitor the location and behavior of the target pet cats, there are concerns regarding distress and safety for things like collars attached to the cats [7]. In this study, leveraging the advancements in computer vision and artificial intelligence technologies, we propose a cat identification system for monitoring the health of pet cats.

We introduce a novel two-step system that relies on machine learning (ML) methods, specifically integrating an object detector and a classification module. We focus on identifying the optimal combination within our classification module, aiming to achieve the highest accuracy in individual cat identification. Specifically, this study focuses on monitoring defecation and urination activities, which serve as clues for diagnosing the health of cats, and conceptualizes and experiments with a cat identification system in litter boxes. One of the common diseases that can occur in domestic cats is feline lower urinary tract disease (FLUTD), which refers to diseases affecting the cat’s bladder or urethra, presenting symptoms such as pollakiuria, periuria, stranguria, and hematuria [4,8]. If a cat shows pollakiuria, we may suspect diseases such as cystitis or urinary stones. Meanwhile, diseases such as feline chronic colitis can affect a cat’s defecation frequency [9]. In multi-cat households, recognizing cat activities in the litter box and determining the frequency of individual cats’ defecation and urination activities are effective in diagnosing these cat digestive and excretory organ conditions.

In the case of multi-cat households, the composition of the cat population may change over time—whether due to the adoption of new cats or other factors. Additionally, cats undergo significant morphological transformations as they transition from kittens to adults—a process that occurs within a relatively short span of time. Given the complexity of this pattern and its potential impact on cat identification, we design an algorithm specifically tailored to make our model highly adaptive to these appearance shifts. This adaptability is crucial not only for recognizing evolving cat features but also for addressing the dynamic environments in which cats reside. For instance, the location and lighting conditions of a litter box can vary. Our retraining mechanism ensures that the monitoring system remains updated, factoring in these variable environments and the changing appearances of cats. This continuous recalibration empowers our system to consistently deliver precise identification.

Furthermore, we compile and utilize a cat body dataset obtained through monocular cameras placed in litter boxes. This dataset includes bounding box labels for both the cat’s face and body. For the body bounding box, there might be instances where the face is not present, resulting in a higher degree of freedom for specific entities. Despite using these body labels for training, our model exhibits good identification accuracy. This success not only underscores the robustness of our ML algorithms, but also highlights the potential of leveraging diverse data sources to enhance diagnostic precision.

One of the main contributions of this study is the determination of the optimal combination for the classification module of our proposed monitoring system. The usability of the model is enhanced by training it with a body image dataset that captures the entire cat body, offering a higher degree of freedom, rather than focusing solely on the cat’s face. In addition, we propose a model-retraining algorithm to ensure consistent performance, especially for cats whose appearances may change rapidly.

The remainder of this paper is organized as follows. Section 2 reviews previous research relates to animal identification and model retraining. Section 3 outlines our method for investigating the best approach to cat identification across the entire architecture. Section 4 presents the experimental results and identifies the optimal combination for the classification module. Section 5 and Section 6 provide a discussion and summary of this study, along with our contributions and suggestions for future research.

## 2. Related Works

### 2.1. ML-Based Identification Systems

Recent studies have proposed deep learning (DL)- and ML-based architectures for the individual identification of various animal species. Hou et al. [10] conducted research on recognizing the faces of 25 giant pandas (*Ailuropoda melanoleuca*) using an architecture composed of a convolutional neural network (CNN)-based model, the Visual Geometry Group Network (VGGNet) [11], with a softmax layer as the classifier. Hitelman et al. [12] designed a biometric identification system for sheep (*Ovis aries*) by employing a two-step approach involving detection and classification. They utilized a Faster R-CNN [13] for sheep-face detection, and compared seven CNN classification models trained with the ArcFace loss function [14]. Schofield et al. [15] focused on the facial recognition of wild chimpanzees (*Pan troglodytes verus*) using a single-shot detector (SSD) model [16] as the detector and a VGG-M [17] based network for identification. Clapham et al. [18] concentrated on the facial recognition of 132 brown bears (*Ursus arctos*) by adopting Schroff et al.’s [19] approach and implementing the overall structure using the dlib toolkit [20]. They utilized a sliding-window-based CNN and an ensemble of regression trees for face detection and alignment in object detection. Following face reorientation and cropping, they generated bear face embeddings via ResNet-34 [21]. Identity classification was carried out using a linear support vector machine (SVM), and the encoding model was trained utilizing pairwise hinge loss.

Similarly, in this study, we explore a CNN-based detector and feature extractor to recognize cats. However, unlike previous studies, we focus on a system that identifies cat body images rather than only cat faces. For the detection model, we utilize YOLOv4 [22], which is known for its fast and accurate performance among the CNN-based detectors. For the classification module, we design an architecture based on [19]. We use a CNN model, EfficientNetV2 [23], as the feature extractor of our architecture. EfficientNetV2 is a powerful CNN model that specifically focuses on efficiency in terms of parameters, floating-point operations or FLOPs, and training speed. Subsequently, ML-based classifiers are used to identify individual cats. Table 1 summarizes existing DL- and ML-based animal identification methods.

### 2.2. Model Adaptation for Concept Drift

In ML and data science, the evolving appearance of pet cats as they mature and grow can be equated to a form of concept drift. Concept drift refers to the phenomenon where the statistics of a target variable in data-based learning models change after the initial training [24]. In various real-world domains, shifts in the data distribution can trigger concept drift, which degrades the model performance over time.

Typically, concept drift adaptive models are initially trained on the target variable, detect drift from the classification accuracy or the statistical characteristics of the data distribution, and are subsequently retrained to accommodate the detected drift. For example, the drift detection method [25] analyzes the error rate of input data to detect abrupt drifts. In the case of adaptive windowing [26], it assumes that there is no change in the distribution of the input data and the combined mean of the two sub-windows is compared when new data are used.

In animal monitoring, the impact of concept drift is significant if there is no control over the external environment or the subjects being monitored. Moallem et al. [27] proposed a system for detecting wild birds by using a two-stage deep neural network pipeline. With a particular focus on the changes in the background of the data, they suggested retraining the model if the average of the images collected throughout the day deviated from the mean images from any single day in the training set.

We propose a periodic retraining method without drift detection. With this method the model continuously adapts to the concept drift stemming from cat class changes, appearance changes in a cat’s life cycle, and environmental factors like lighting or backgrounds. Considering the memory efficiency of the recognition server, the number of embedding vectors is fixed when retraining the ML-based classifier. This iterative refinement ensures that the model remains robust and accurate, capturing the intricacies of a cat’s evolving appearance and the dynamic conditions under which they are observed.

## 3. Proposed Method

### 3.1. Cat Identification Architecture

**Figure 1:**
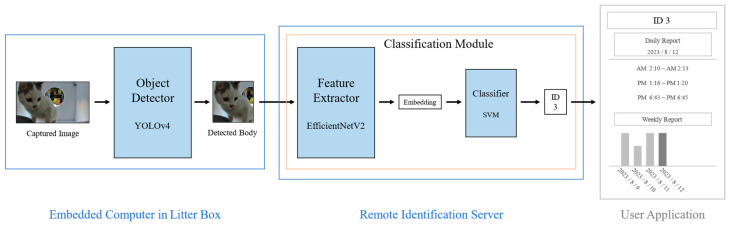
Overall architecture of the proposed cat identification model.

Our cat identification architecture comprises two key components: the object detector in an embedded computer within the cat litter box, and the classification module hosted on the server. The overall architecture for our system is shown in Figure 1.

When the cat enters the litter box, a RGB camera equipped with an embedded computer detects its defecation activity and records it as a video. The object detector in the litter box focuses on capturing the entire body of the cat during these activities. When a series of body images for an activity are detected, they are transmitted to a remote server for identification of the given cat body images.

**Figure 2:**
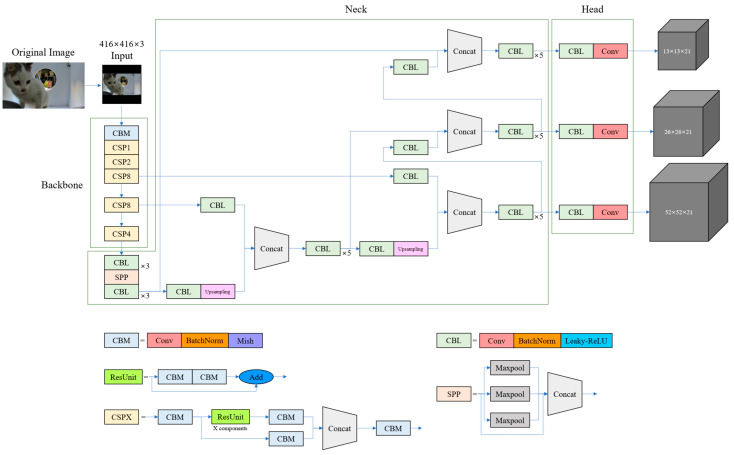
YOLOv4 network structure for cat body detection.

We use YOLOv4 for the detector model, which enables accurate real-time detection within an embedded computer. The structure of the YOLOv4 network is illustrated in Figure 2. YOLOv4 incorporates various optimizations into the YOLOv3 [28] model. Notably, YOLOv4 adopted the cross-stage hierarchy approach of the cross stage partial network (CSPNet) [29] to change the Darknet53 from YOLOv3 to CSPDarknet53. This approach significantly reduced the computational cost of each layer in the backbone, resulting in improved performance during training and inference. Additionally, YOLOv4 leveraged the structures of the spatial pyramid pooling network (SPPNet) [30] and the path aggregation network (PANet) [31] in its neck module to increase the receptive field and enhance detection performance by augmenting different backbone paths. Based on the exceptional performance and learning efficiency of YOLOv4, we adopt it as the detector in our cat identification model.

**Figure 3:**
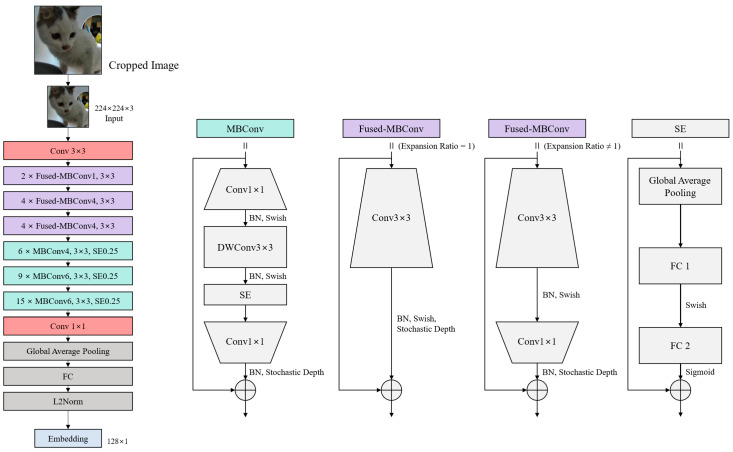
Modified EfficientNetV2-S network structure for the feature extractor in the classification module.

In the identification server, the final inference is achieved through with two model components: a feature extractor and a classifier. When the feature extractor receives a body image as input from the litter box, it extracts an embedding vector from the given image. To accomplish this, the feature extractor employs EfficientNetV2. The overall structure of the feature extractor is illustrated in Figure 3. EfficientNetV2 is an improved version of the EfficientNet [32] model, which was derived from a mobile neural architecture search network (MnasNet) [33] and utilizes a neural architecture search (NAS) [33,34] to determine the optimal model compound scaling dimensions (depth, width, and resolution). By incorporating mobile inverted bottleneck convolution (MBConv) blocks with squeeze and excitation blocks, it reduced computational complexity while enhancing the performance. EfficientNet demonstrated significantly higher performance with a much smaller number of parameters than traditional CNN models. EfficientNetV2 focused on enhancing training efficiency. This was achieved by using Fused-MBConv blocks early in the model, which replaced depth-wise 3 × 3 convolutions with regular 3 × 3 convolutions, reducing the overhead associated with depth-wise convolution GPU operations. Additionally, rather than employing the traditional compound scaling method that uniformly scales the model size, EfficientNetV2 utilized non-uniform scaling in the later stages of the model, placing more emphasis on scaling to find a more efficient model structure for training. These advancements in EfficientNetV2 have contributed to its superior efficiency and performance compared to its predecessors. Considering the limited server resources and the need for frequent retraining, we select EfficientNetV2 for its lightweight characteristics and rapid training capabilities.

This network extracts a 128-dimensional embedding, following the architecture design outlined in [19], which is then fed into the ML-based embedding classifier. The classifier uses these feature vectors as inputs and subsequently determines the class of cats present in each input image. Once a class is determined by the classifier, the server stores and aggregates data regarding the cat’s activity. A user can check the daily or weekly litter box usage status for each pet cat, along with the stored videos. By understanding the frequency and condition of defecation and urination, the user can gauge the state of the cat’s digestive and excretory organs.

### 3.2. Classification Module Adaptation

Within the litter box, the overall environment observed by the camera can continuously change over time. The background may shift due to factors such as relocating the litter box or altering the lighting conditions at the litter box location. Additionally, foreground distribution can undergo significant changes owing to changes in the composition of cats or individual transformations in the appearance of the cats themselves. In such dynamic situations, retraining vision DL models and embedding-based ML models is crucial for consistently maintaining high identification performance.

In this context, we design a classification module to effectively adapt to changes in the composition or appearance of a user’s pet cats through an interactive scenario between the user and the server. This allows the server to continuously update and refine its identification capabilities based on user input and feedback, ensuring the accurate and personalized identification of pet cats over time.

**Figure 4:**
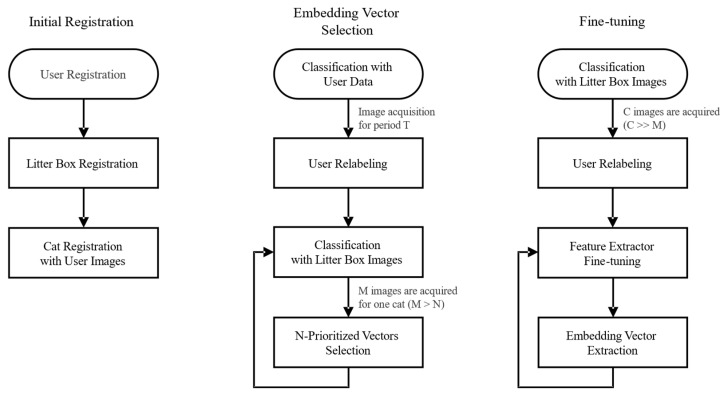
Model retraining algorithm for the feature extractor of the classification module.

Initially, a user registers the information of the litter box and cats on the server. Since the model classifier cannot make predictions from litter box images without any cues, a set of images taken by the user for each cat is also transmitted to the server. After registration, a two-step retraining process is designed, comprising embedding vector selection and fine-tuning. A flowchart illustrating these steps is shown in Figure 4.

As the server continues to collect images over a specific period (T), the user verifies whether the cat identification is properly conducted and relabels the classes of these images. At this time, the user labels one video in which the cat entered and exited, and all images corresponding to the labeled video are labeled at once. Then, the model classifier is retrained with these relabeled litter box images to obtain more accurate identification. As a certain number (M) of new images for each cat is gathered, a mean vector is calculated using both the existing embedding vectors and the collected vectors. This mean vector serves as a representation of the cat’s characteristics. To optimize the training data, the embedding vectors closest to this mean vector are selected (N-prioritized vectors), while the remaining vectors are discarded. Through this approach, we can remove outlier data for a specific class and stabilize the amount of training data for that class, thereby maintaining memory efficiency on the server. In addition, when a new cat is registered, the embedding vector selection for that cat starts by learning the user’s image for the cat and importing the images during period T. If a user deregisters a specific cat, the embedding vector for that cat is deleted.

Once a sufficient number (C) of images for all cats are collected, the user again relabels the images. The feature extractor is fine-tuned using the relabeled dataset. A fine-tuned feature extractor is used to extract new embedding vectors from the training sample images. By retraining the extracted embedding vectors back into the classifier, the model can adapt to changes in the appearance of pet cats within a particular household.

This iterative and interactive approach ensures the continuous enhancement and refinement of the system performance, as it continuously adapts to changes in the configuration and individual appearance of a user’s pet cats, and to changes in the external environment. By leveraging the new data and user feedback, our retraining process aims to achieve the accurate and personalized identification of pet cats over time, ultimately improving the overall efficiency and effectiveness of the system.

## 4. Results

### 4.1. Environment

The overall experiment is performed on a desktop computer equipped with an Intel^®^ Core™ i7-12700KF and 16.0 GB RAM. The architecture is trained using an NVIDIA RTX A6000. All source codes, including training and testing, are implemented employing Python 3.8 and PyTorch 11.3 libraries with the CUDA toolkit on Ubuntu 21.04.

### 4.2. Dataset

To demonstrate the proposed cat monitoring system, we first create our own cat body dataset. Using customized litter boxes (515 × 695 × 475 mm, with a hole in front) with a monocular camera, we gather diverse cat image data by recording videos of their activities. For experiments on the identification module, bounding boxes for the face and body are labeled and used for the collected images. The litter boxes are positioned in various environments such as cat cafés, streets, and shelters.

**Figure 5:**
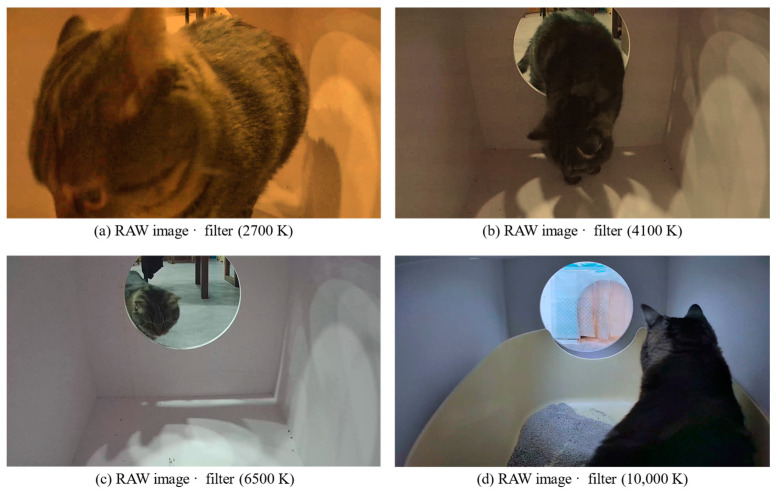
Color temperature diversification examples. (**a**) Image on warm white (2700 K); (**b**) image on natural white (4100 K); (**c**) image on cool white (6500 K); and (**d**) image on LED light (10,000 K).

To make the architecture robust to changes in the color temperature, we diversify the dataset by applying four color filters: warm white (2700 K), natural white (4100 K), cool white (6500 K), and LED light (10,000 K) by 1:1:1:1, as shown in Figure 5. It should be noted that when we acquire the data, we assume that the color temperature environment was cool white. So there is no difference before and after passing through the filter for cool white.

The dataset comprises 8934 RGB Full HD images of 36 cats. We divided the dataset into 8:2 ratios using stratified sampling for model training and evaluation.

### 4.3. Classification Module Training

A metric learning technique is used to enable the feature extractor to learn the similarity of data in the embedding space. We train the feature extractor using triplet loss [10]. The loss function employed in this study demonstrated excellent performance in facial recognition tasks. It utilizes a technique that learns the structure of the feature representation by separating the positive pairs and negative pairs. This approach aims to enhance the discriminative power of feature embeddings, allowing the model to effectively distinguish between similar and dissimilar instances, thereby improving the accuracy and effectiveness of the recognition system.

The overall training process comprises three stages; each stage utilizes semi-hard, hard, and hardest triplets. The margin for calculating the loss is set at 0.2. We also add a global orthogonal regularization term [35] to the loss function, to spread the features throughout the embedding space. Adam is used as the model optimizer, with the learning rate and momentum parameters *β*_1_ and *β*_2_ being set to 0.001, 0.9, and 0.999, respectively.

**Figure 6:**
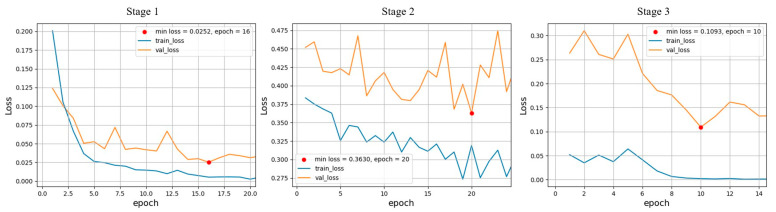
Loss graphs of each training stage for the feature extractor. Each stage utilizes semi-hard, hard, and hardest triplet minings.

To train the feature extractor, 7162 images of 36 cats are used, which are split into 8:2 ratios for training and validation. If the validation loss does not decrease for 5 epochs during a stage, the training for that stage is stopped early; otherwise, it continues for up to 100 epochs. The loss graph for each training stage is shown in Figure 6.

Once the feature extractor is trained, we obtain the embedding vectors from all training and validation data and use them to fit the classifier. By comparing the identification accuracies of different ML classifiers, we select the one with the highest accuracy as the final model classifier.

### 4.4. Evaluation Metrics

In this experiment, we assess the classification module’s performance using several evaluation metrics, including accuracy, confusion matrix, receiver operating characteristic (ROC) curves, and precision-recall (PR) curves.

The accuracy is the percentage of samples that the classifier classifies from a given sample into the correct class:

Accuracy = Correct predictions/All predictions.(1)

The confusion matrix visualizes the performance of the classification algorithm and comprises true positives (TP), false negatives (FN), false positives (FP), and true negatives (TN). TP is the number of samples which accurately classify a category of interest as a category of interest. FN is the number of samples that misclassify a category of interest as not a category of interest. FP is the number of samples that misclassify non-interest categories as interest categories, and TN is the number of samples that accurately classify non-interest categories.

The ROC curve expresses the relationship between the true positive rate (TPR) and false positive rate (FPR) as practice values for trainees, which are calculated as follows:

TPR = TP/(TP + FN),(2)

FPR = FP/(FP + TN).(3)

The PR curve expresses the relationship between recall and precision as a threshold for class-specific probability; recall and precision are calculated as follows:

Recall (R) = TP/(TP + FN),(4)

Precision (P) = TP/(TP + FP).(5)

### 4.5. Experimental Results

#### 4.5.1. Performance Comparison with Different Classifiers

**Figure 7:**
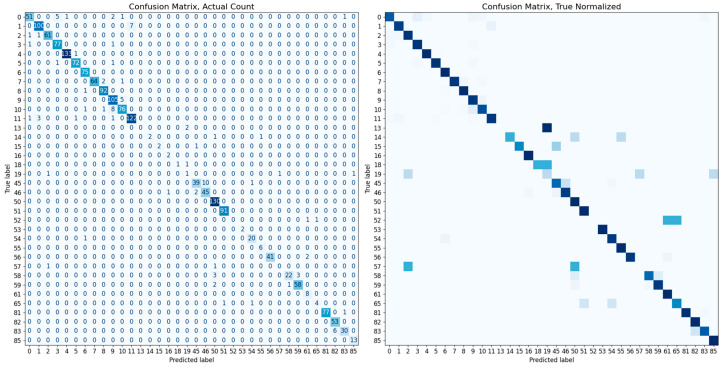
The confusion matrices for the SVM classifier with a linear kernel. Each left and right matrix shows the confusion matrix without/with normalization by the number of test set images in each class. The color of each cell in the matrix represents the number of images or normalized ratio, with darker colors corresponding to larger values.

**Figure 8:**
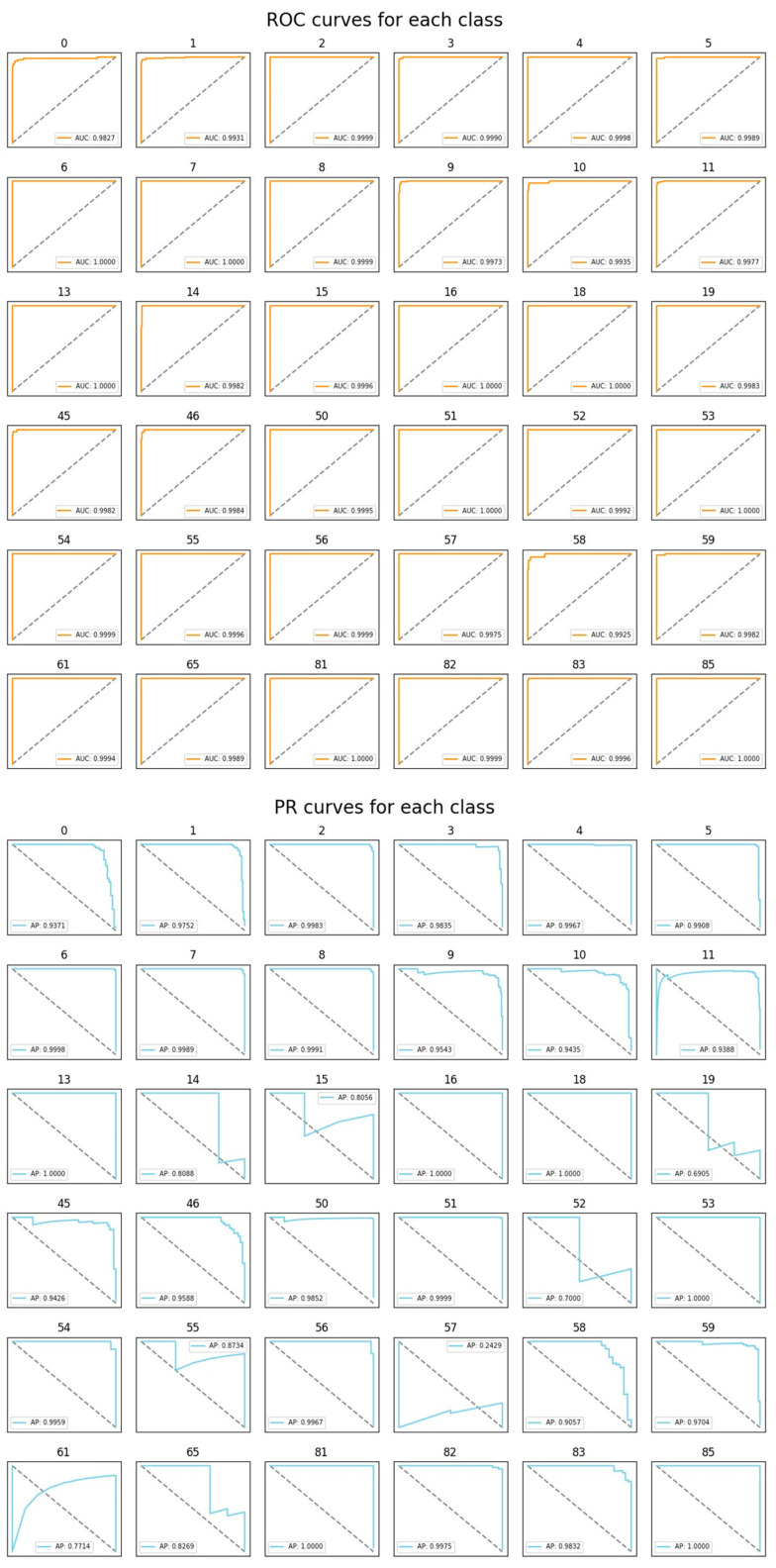
The ROC and PR curves for the SVM classifier with a linear kernel. For each class, AUC (area under ROC curve) and AP (average precision) values are indicated.

**Figure 9:**
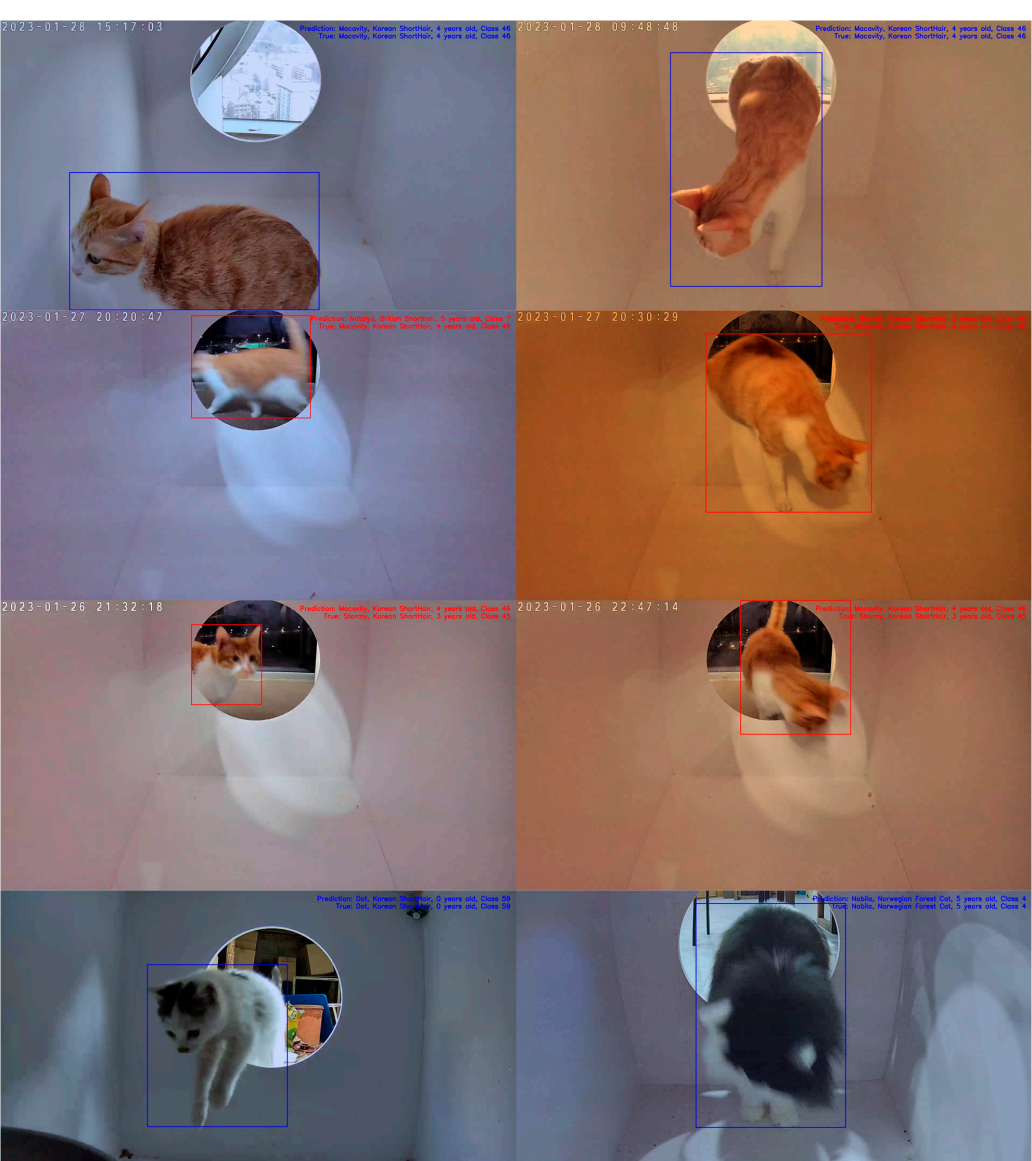
Examples of identification results for specific cat images of our dataset. Each row corresponds to TP, FN, FP, and TN from the top to the bottom. For each image, if the identification result is correct then the color of bounding box and information are displayed in blue; otherwise, they are displayed in red.

In this study, the performance of various classification methods is evaluated using a test dataset comprising 1772 images of 36 different cats. Each test image is classified based on its embedding vector, using a complete set of embedding vectors from the training dataset of 36 cats. We examine classifiers such as K-nearest neighbors (KNN), random forest, and SVMs. The identification accuracies of the classifiers are listed in Table 2. Notably, the SVM classifier equipped with a linear kernel achieves the highest identification accuracy of 94.53%. Other performance metrics, such as the confusion matrix, ROC curves, and PR curves, are illustrated in Figure 7 and Figure 8. The additional inference results are presented in Figure 9.

#### 4.5.2. Performance Comparison with Different Data

**Figure 10:**
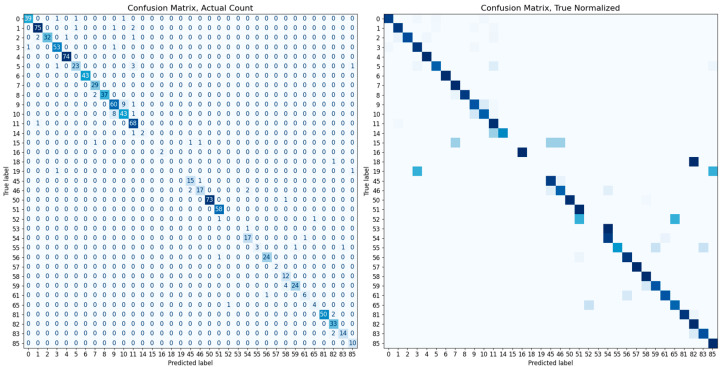
The confusion matrix evaluated on the cat body dataset. Each left and right matrix shows the confusion matrix without/with normalization by the number of test set images in each class. The color of each cell in the matrix represents the number of images or normalized ratio, with darker colors corresponding to larger values.

**Figure 11:**
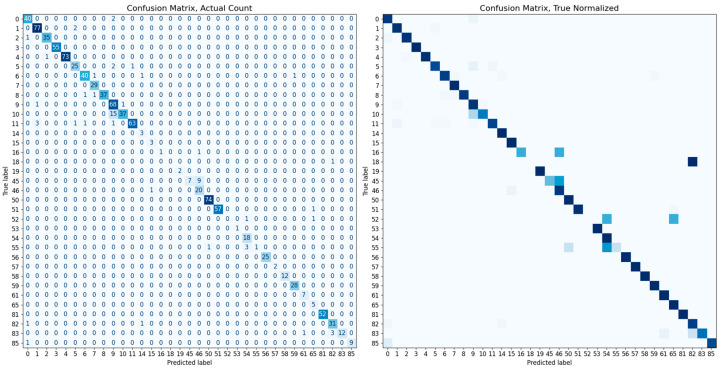
The confusion matrix evaluated on the cat face dataset. Each left and right matrix shows the confusion matrix without/with normalization by the number of test set images in each class. The color of each cell in the matrix represents the number of images or normalized ratio, with darker colors corresponding to larger values.

To confirm the superiority of our system’s performance when using cat body labels, we conduct an additional experiment to compare the performance when identifying cats using only the cat face, similar to other existing animal recognition methods. Within the complete image dataset, some images comprise only body without a face. For a fair performance comparison, we use 5115 images from 35 types after excluding those without faces; for class 13, no images include faces. Consistent with the previous experiment, the dataset is divided into an 8:2 ratio using stratified sampling for model training and evaluation. The training of the classification module is executed in the same manner as described in Section 4.3., and an SVM classifier with a linear kernel is used for the final accuracy measurement. Both models show a high performance of over 90%, and even with a higher degree of freedom such as the body, the performance difference is minimal at about 0.69%. The overall identification accuracy and confusion matrix for each dataset are presented in Table 3 and Figure 10 and Figure 11.

## 5. Discussion

In this study, we train and evaluate the proposed algorithm using a body dataset of 36 different cat species. During evaluation, we compute the identification accuracy for the test dataset with all 36 class embeddings trained in the classifier. During the training and evaluation phases, we observe a high identification accuracy, which is a promising indicator of the system’s potential effectiveness. In particular, the combination of EfficientNetV2-S with the SVM classifier yields outstanding results with an impressive accuracy of 94.53%.

Notably, even in scenarios where the data could be confusing, such as images that include not only the cat’s face but also its body, our architecture demonstrates only a slight performance decrease of approximately 0.69% compared with the face recognition model. This success underscores the ability of the model to handle diverse and complex visual inputs.

## 6. Conclusions

We focus on the integration of a neural-network-based feature extractor with existing ML-based classifiers to develop a cat monitoring system. Additionally, we propose a model retraining algorithm with the aim of enabling the DL-based model to adapt seamlessly to the dynamic appearance changes that occur during a cat’s life cycle and variations in the camera environment.

To validate the monitoring performance of the proposed system, we collect a pet cat body dataset through a monocular camera inside the litter box. Instead of the face commonly used for animal identification, our system uses the entire body for identification (where the face may not be included), making the scenario correspond to a more challenging task. We perform testing on the classification module and compare the performance of various ML-based classifiers. The combination of EfficientNetV2-S and the SVM classifier demonstrates a high identification accuracy of 94.53% on the entire cat body dataset, and the identification performance receiving a body dataset with face is only about 0.69% lower than when receiving a face dataset. This indicates that our cat body identification system has high applicability in monitoring the defecation and urination activities of pet cats in multi-cat households.

In future research, there are several avenues for further improvement. Exploring different stage combinations, optimizers, and advanced loss functions during training can lead to performance enhancement. In addition, the evaluation and development of various models for the detector part of the system will contribute to a more comprehensive and refined architecture. Meanwhile, a mathematical validation of the model and retraining method presented in this study is required. Especially, since a mathematical analysis of the recognition system has not been addressed, there is a need to ascertain the solution for the mathematical model of this research system and its stability [36,37]. Lastly, in addition to the vision-based identification system, it is an important consideration in the future to analyze the cat’s defecation and urination status more precisely and comprehensively by measuring the cat’s litter box activity time or integrating more diverse sensors, such as weight, humidity and pH of urine and feces.

The cat litter box monitoring system developed in this study contributes to managing the urinary health of individual cats in multi-cat households. Moreover, the identification architecture of this system is not limited to cat litter boxes and can be expanded to other situations, aiding in personalized monitoring and diagnosis for multi-cat households.

## Figures and Tables

**Table 1:** A summary of existing animal identification methods. (OD = object detector, CL = classifier).

Method	Region of Interest	Architecture
Hou et al. [10]	Face	CL: VGGNet [11]
Hitelman et al. [12]	Face	OD: Faster R-CNN [13] CL: CNN-based models
Schofield et al. [15]	Face	OD: SSD [16] CL: VGG-M [17]
Clapham et al. [18]	Face	OD: CNN-based model CL: ResNet-34 [21] + SVM
Ours	Body	OD: YOLOv4 [22] CL: EfficientNetV2 [23] + ML-based models

**Table 2:** Identification accuracy evaluated on the test dataset with different classifiers.

Classifier	Accuracy
KNN (K = 3)	94.24%
KNN (K = 5)	94.41%
KNN (K = 7)	94.19%
Random forest	94.13%
SVM (RBF kernel)	94.41%
SVM (Linear kernel)	94.53%

**Table 3:** Identification accuracy evaluated on the cat body dataset and cat face dataset.

Dataset	Accuracy
Cat body with face	93.08%
Cat face only	93.77%

## Data Availability

Data sharing is not applicable to this article.
